# Single-cell Raman and mass spectrometry analysis to probe cellular heterogeneity in tamoxifen uptake and metabolism

**DOI:** 10.1007/s00216-025-06058-w

**Published:** 2025-08-14

**Authors:** Congrou Zhang, Yasmine Abouleila, Sylvia Le Dévédec, Thomas Hankemeier, Arno Germond, Ahmed Ali

**Affiliations:** 1 Metabolomics & Analytics Centre, LACDR, Leiden University, Leiden, the Netherlands; 2 Riken Biodynamics Research Center (BDR), Kobe, Japan; 3 Drug Discovery & Safety, LACDR, Leiden University, Leiden, the Netherlands; 4 Quality of Animal Product, INRAE, Saint-Genès-Champanelle, Centre Auvergne Rhône-Alpes France

**Keywords:** Single-cell mass spectrometry, Raman spectroscopy, Tamoxifen, Cellular heterogeneity

## Abstract

Current drug discovery is limited by the lack of single-cell data on drug uptake, metabolism, and effects, as population-level methods obscure cellular heterogeneity. While single-cell RNA sequencing has revealed drug resistance mechanisms, it cannot simultaneously measure drug concentrations and cellular responses. Raman spectroscopy probes single-cell drug effects but lacks sensitivity for drug or its metabolite quantification, whereas single-cell mass spectrometry (MS) offers high sensitivity but consumes samples, preventing repeated measurements. Integrating Raman spectroscopy with MS enables simultaneous assessment of cellular states and drug metabolism. However, existing studies are limited by small sample sizes and single drug concentrations. We employ a combined single-cell Raman and mass spectrometry (Raman-MS) approach to investigate variability in drug uptake, metabolism, and effects in HepG2 liver cancer cells. The cells were exposed to three concentrations of tamoxifen, after which we quantified the heterogeneity in tamoxifen and its hepatotoxic metabolites. This validates the potential of single-cell analysis for advancing drug discovery and cancer research. Our results indicated that tamoxifen induces concentration-dependent metabolic changes in single liver cancer cells, as revealed by Raman spectroscopy and mass spectrometry. The findings highlight a potential threshold concentration beyond which cellular integrity is compromised, underscoring the importance of single-cell approaches for understanding drug uptake, metabolism, and therapeutic heterogeneity.

**Graphical Abstract:**

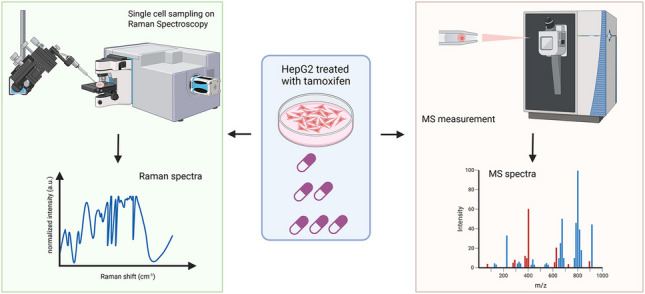

**Supplementary information:**

The online version contains supplementary material available at 10.1007/s00216-025-06058-w.

## Introduction

The lack of single-cell data on drug uptake, metabolism, and effect is hampering current drug discovery efforts [1], especially for anti-tumor drugs, where a substantial proportion fail during phase II clinical trials [2]. Since drugs interact with live cells, which are highly dynamic, it is vital to quantify drug and its metabolites at both local and population levels. However, current studies rely on population-level analytical methods in drug discovery which averages across heterogenous populations [3–5]. Consequently, potentially clinically relevant subpopulations and cellular heterogeneity in general is masked [6]. The result of using bulk-level analyses in drug discovery is that the level and impact of cellular heterogeneity on drug uptake, metabolism, and effect are unclear. Currently, there is research on quantifying the heterogeneity of cellular interaction with drugs at the single-cell level, or investigating how these interactions change with increasing concentrations of the drug remains limited [7, 8].

Several studies on cancer cells have attempted to address this gap by studying the impact of single-cell heterogeneity on drug interactions using single-cell RNA sequencing primarily. Single-cell RNA sequencing revealed the dynamics of the stress response at increasing anti-cancer drug concentrations at the single-cell level [9]. In addition, it was used to study drug-resistant subpopulations of cancer cells and to identify molecular mechanisms of said resistance that are otherwise masked with traditional population-level methods [10]. Other studies used single-cell RNA sequencing to examine the intratumor heterogeneity of a pair of primary renal cell carcinomas and their lung metastases. They found that activation of drug target pathways indicated considerable differences between primary and metastatic sites, as well as between individual cancer cells within each site. These results show that single-cell RNA sequencing can not only elucidate transcriptional heterogeneity during metastatic progression but can also be used to design optimized combinations of targeted drugs against metastasis [11, 12]. Despite the utility of RNA sequencing, the simultaneous monitoring of cellular responses to drugs at the single-cell level and the drug concentration in single cells is beyond its capabilities. Therefore, to unravel single-cell drug response, new technologies are needed to screen for the drug effects and simultaneously measure concentrations of the drug and its metabolites at the single-cell level.

A promising technique that can help probe cellular response to drugs on the single-cell level is Raman spectroscopy [13]. Previous studies have shown how Raman can identify organelles and metabolic shifts in cells in different cellular processes such as apoptosis, lipid production, cell differentiation, and reprogramming [14–17]. By measuring highly abundant cellular biomolecules (i.e., lipids, proteins, DNA, and RNA features) Raman spectroscopy was able to demonstrate that changes in the relative abundance of these biochemical compounds correlate with differences in cancer proliferation [14]. More importantly, Raman spectroscopy can show the effect of increasing concentrations of anti-cancer drugs on the cancer cells’ genome [18, 19]. Therefore, Raman spectroscopy can be used to describe the current cellular state at the single-cell level and to study the effects of drugs in single cells. Despite its utility, the current detection limits for drugs remain at the milligram level, which limits its sensitivity for identifying trace quantities of drugs and their metabolites at the single-cell level [20]. Therefore, while it is able to determine the drug effect on a single-cell level, it needs to be combined with other techniques to quantify drug uptake and metabolism, such as mass spectrometry [21].

Single-cell mass spectrometry (MS) can provide detailed molecular information quantitatively due to its high sensitivity. It was used successfully to investigate the subtle changes in drug response and to identify potential biomarkers indicative of therapeutic effects or adverse reactions [6]. Single-cell nano-LC–MS was used by Nakatani et al. [22] to classify HeLa cells into three distinct cellular subclasses based on their metabolic differences, which provided the basis for refining anti-cancer treatment. On the other hand, Masujima et al. [23] reported a direct infusion-based live single-cell MS method in which individual cells are first collected into a coated capillary with an internal standard in an ionized solvent, followed by a direct MS injection. Despite their promise, mass spectrometry-based methods consume the samples during measurements; therefore, the limited sample volume of single cells precludes repeated or prolonged measurements. This makes measuring the drug effect as well as quantifying it on the single-cell level challenging due to the sample destruction required. However, combining single-cell MS with another non-destructive technique such as Raman can enable the accurate determination of the cellular state and simultaneous quantitative measurement of drug metabolites. To challenge this, several analytical platforms have emerged that attempted to combine MS with non-destructive methods. High-performance liquid chromatography (HPLC) in conjunction with infrared ion spectroscopy was used for the identification of positional isomers of hydroxy-atorvastatin. Using this method, the two hydroxylated metabolites were separated using HPLC and then identified by their IR spectra [24]. In addition, Eric J. Lanni et al. [25] combined secondary ion mass spectrometry and confocal Raman microscopy, analyzing the chemical composition of broad molecular constituent classes in Pseudomonas-derived biofilms. Finally, Ali et al. used Raman spectroscopy and mass spectrometry to screen for drug effects on the single-cell level. However, this study suffered from a limited number of samples, and in studies where cells were exposed to drugs, a single concentration of the drug was used, therefore making it challenging to draw biological conclusions [21]. A key unanswered question in these studies is the degree of the variability of drug uptake in single cells with different concentrations of the spiked drug.

To answer the aforementioned question, we aim to determine the heterogeneity and overall trend in drug uptake, metabolism, and effect in single cancer cells using a combined single-cell Raman-MS approach with multiple drug concentrations. By incrementing the drug concentration, we aimed to observe and quantify the variability in drug uptake and metabolism of the anti-cancer drug tamoxifen in HepG2 cells. HepG2 cells were chosen because they metabolize tamoxifen into its two pharmacologically active hepatotoxic metabolites, namely 4-Hydroxytamoxifen (4-OH-tamoxifen) and N-desmethyltamoxifen. Using this integrated Raman-MS methodology, we aim to understand the heterogeneity of drug uptake, metabolism, and response of individual cells and to provide evidence and new ideas for the development of an integrated platform capable of monitoring single-cell pharmacokinetics and pharmacodynamics.

## Materials and methods

### Cell culture

HepG2 cells were used in this study as the cell model. Cells were obtained from the RIKEN Biological Resource Center (BRC) cell bank. Frozen cells stored in a liquid nitrogen tank were thawed and preheated in a 37 °C water bath (Thermo minder, Taitec Co., Saitama, Japan) for 1 min. The cells were then cultured in medium containing Dulbecco’s modified Eagle’s medium (DMEM, Sigma-Aldrich, Minnesota, USA) supplemented with 10% fetal bovine serum (FBS) obtained from Hyclone Laboratories, Hyclone, Utah, USA, and 0.1% penicillin–streptomycin (Nacalai Tesque, Kyoto, Japan). Cells were kept in a humidified incubator (MC0-19A1C, Sanyo Electric Co., Osaka, Japan) at 37 °C with 5% CO_2_ for 2 days. All cultured cells were synchronized to reach 50–60% confluency prior to drug treatment to minimize variations. In each experiment, cells were cultured in 35 mm glass-bottomed grid dishes (Matsunami, Osaka, Japan) pre-coated with rat tail collagen coating solution (Cell Applications Inc., San Diego, USA) and then incubated for 24 h before drug treatment.

### Drug treatment

In each experiment, cells were divided into four subgroups, one is an untreated group that was used as control, and three are tamoxifen-treated groups. To spike the cells with tamoxifen, cells were washed twice with PBS. Then, tamoxifen (Sigma-Aldrich, Minnesota, USA) dissolved in dimethyl sulfoxide (DMSO, Nacalai Tesque, Kyoto, Japan) was mixed with the culture medium so that the final volume of the drug-treated group was 2 mL. The previous study [21] shows that cells treated with 10 μM of the tamoxifen exhibit differences in both Raman and MS spectra. To investigate cell heterogeneity across different drug concentrations, the concentrations for the three drug-treated groups were set to 5 μM, 10 μM, and 20 μM. A corresponding volume of solvent (DMSO) was mixed in the medium as a control for the effect of DMSO. The incubation was continued for 24 h in both groups.

### Raman spectral imaging and spectral preprocessing

Prior to observation, cells were extracted from the incubator and underwent a double wash to remove tamoxifen in the medium. The medium was replaced with warmed FluoroBrite DMEM from Thermo Fisher Scientific (Massachusetts, USA). FluoroBrite DMEM was chosen due to its compatibility with Raman spectroscopy, providing a similar background level as PBS while offering enhanced nutrition for the cells. The samples were positioned within a heated microchamber (ibidi, Munich, Germany) affixed to a motorized microscope stage (BIOS-L101T-S, Opto-Sigma, Tokyo, Japan). The microchamber maintained a 5% CO_2_ environment and a constant temperature of 37 °C during the measurements. Raman spectral assessments were conducted using a home-made confocal scanning Raman microscope described in a previous study [26]. Employing a line illumination system, a 532 nm diode-pumped solid-state laser (Ventus, Laser Quantum, UK.) was directed to live cells a few micrometers above the optical glass surface through the objective lens (NA: 0.95, UPL40, Olympus, Tokyo, Japan). The spectra were captured with a cooled CCD camera (PIXIS BR400, Princeton Instruments, New Jersey, USA) mounted on a polychromator, utilizing a 1200 g/mm grating to optimize spectral resolution in the fingerprint region (600 to 1800 cm^−1^). The spatial resolution of the system was approximately 300 nm, with a spectral resolution of 1 cm^−1^.

Living cells were subjected to a 10-s exposure with a laser intensity of 2.4 mW/μm^2^. A single-line exposure was employed, and data from pixels corresponding to each single cell were averaged to minimize the time gap between Raman and MS measurements of the same cell. Hyper-spectral images underwent processing using in-house algorithms for cosmic-ray removal, background subtraction, baseline correction, and vector normalization, as detailed in a previous study [26]. A total of 202 spectra from single cells were acquired. Immediately after spectral measurements of each cell, the individual cell was sampled using coated glass micropipettes for subsequent mass spectroscopy measurements.

### Sampling for mass spectrometry from single cells

Following Raman measurements, live cells were picked using a glass micropipette with a 5 μm pore size. In total, 152 treated and 50 untreated cells were sampled. Specifically, 49 cells were treated with 5 μM tamoxifen, 49 cells were treated with 10 μM tamoxifen, and 54 cells were treated with 20 μM tamoxifen. The sampling apparatus comprised a micromanipulator connected to a platinum-coated glass micropipette (CT-2, Humanix, Hiroshima, Japan), with the capillary holder connected to a syringe to apply negative pressure during sampling. After measuring a cell of interest using Raman, the capillary holder, guided by the micromanipulator, was lowered, and the cell was sampled by applying negative pressure to the capillary under microscopic observation.

After sampling, 2 μL of ionizing solvent was added to each capillary from the wide end using a pipette attached to an Eppendorf GELoader pipette tip (Eppendorf, Hamburg, Germany). The ionization solvent comprised a mixture of 80% methanol, 10% DMSO, and 0.1% formic acid, all of LC–MS grade from Sigma-Aldrich, Missouri, USA, and 2 ng/mL (5.31 nM) of d5-tamoxifen (Cambridge Isotope Laboratories, Inc., Massachusetts, USA) as an internal standard. The capillary was then connected to a nano-electrospray adapter (nano-ESI), which interfaced with the Q-Exactive mass spectrometer (Thermo Fisher Scientific, Massachusetts, USA) for mass spectrometry analysis.

### Mass spectrometry measurements

Mass spectrometry analyses were performed using a Q-Exactive Orbitrap instrument, calibrated with the Pierce LTQ Velos ESI calibration solution (Thermo Fisher Scientific, Massachusetts, USA). The protocol from Ali et al. [21] was used to set the parameters of the instrument, which are summarized in Table S-1. The experiment was done over the span of 3 days; on each day, all concentrations and controls have been sampled and measured.

### Mass spectrometry data processing and analysis

After mass spectrometry measurements, peak areas were exported from the manufacturer’s proprietary raw format to text files using an in-house script. Each file contained an extracted ion chromatogram (XIC) of the relevant peak, which was imported into Python statistical software for further processing.

Out of the 202 cells sampled, a total of 35 cells could not be analyzed. Four capillaries were broken; therefore, nanospray could not be started, and 31 cell-capillaries were blocked; consequently, a stable spray could not be achieved. Therefore, 167 single-cell data were used for data analysis, including 41 cells from the control group, 37 cells from the 5 μM group, 47 cells from the 10 μM group, and 42 cells from the 20 μM group. All plots were generated using GraphPad and Excel.

### Statistics and machine learning methods

Mean comparisons were performed using ANOVA followed by a Tukey HSD test, and p values of < 0.05 were considered significant.

The 1-dimensional pre-treated spectra were used to train a non-linear Support Vector Machine (SVM) model to test the possibility of identifying each treatment depending on tamoxifen concentrations [27, 28]. The radial basis function (RBF), a Gaussian kernel, was chosen. The training dataset was selected by randomly taking 70% of the spectral dataset. The model was tested using the remaining 30%. This process was iterated 100 times to generate 100 models, and the model outputs were averaged. The output of the SVM classification is a confusion matrix showing the percentages of predicted spectra that are rightly classified when compared to true spectra. These models were computed using Orange data mining software version 3.33 [19, 29].

To identify objectively the spectral frequencies (hereafter called biomarkers) that are associated with each treatment group, we computed the Gini scores (ranging from 0 to 1) to evaluate the contribution of each frequency. To reduce computation time, the average model output was used to calculate the Gini scores. Lower Gini scores indicate better separation between classes, meaning that the variable (here wavelengths) contributes significantly to the classification. Considering the classes C, the Gini score is calculated as

$$Gini=1-\sum\limits_{i=1}^cp_i^2$$

where ${p}_{i}$ is the proportion of elements of class $i$ in the set. Frequencies belonging to different spectral bands are listed in Table 1 with their associated scores.

**Table 1:** List of the peak wavenumbers identified as strongly important to separate treatments. Our identification approach uses the Gini scores calculated using the output of the SVM models. Peaks contributing the most to group separation have higher Gini scores

Wavenumber (cm^−1^)	Molecular assignment	Ref	Gini score	Biomarker strength
647	C–S stretch., C–C twist., associated with protein	[26]	< 0.04	Very strong
752	δ(C–C) Tyr, associated with proteins, cytochrome	[26]	0.048	Very strong
773	Inositol	[30]	< 0.04	Very strong
900	υ(C – O – C)	[26]	0.047	Very strong
971	CH_2_ rock., C–C stretch., α-helix	[26]	0.074	Strong
1000–1002	Phe ring breath., C–C skeletal (protein)	[26]	0.052	Strong
1133	CH, Phe, cytochrome	[26]	< 0.04	Very strong
1173	C–N pyrimidine side, C – H bending, Tyr	[26, 31]	0.085	Strong
1241	T, A, Amide III, CH bend, associated with nucleic acids, proteins, lipids	[26]	< 0.04	Very strong
1334–1340	CH_3_CH_2_ def. of collagen, ATP, associated with nucleic acids, proteins	[26]	0.05	Strong
1403–1422	CH_2_ bending, lipids, Amide bonds of proteins	[30, 32]	0.048	Strong
1441–1450	G, A, CH def., associated with nucleic acids, proteins, lipids, carbohydrates	[26]	< 0.04	Very strong
1541	υ(C ═ = C) stretch., Tyr, proteins	[26]	< 0.04	Very strong
1560	Purines, lipids	[30, 33]	< 0.04	Very strong
1589	C − CO −, C − COO^−^ stretching	[26]	0.067	Strong
1603	C ═ N, aromatic C ═ C associated with tryptophan	[30, 34]	0.11	Strong
1608–1613	υ(C ═ C), Trp, proteins	[26]	0.077	Strong
1650	C═C of proteins	[26]	< 0.04	Very strong

## Results

### Raman spectroscopy detection of drug effects at different concentrations at the single-cell level

**Fig. 1:**
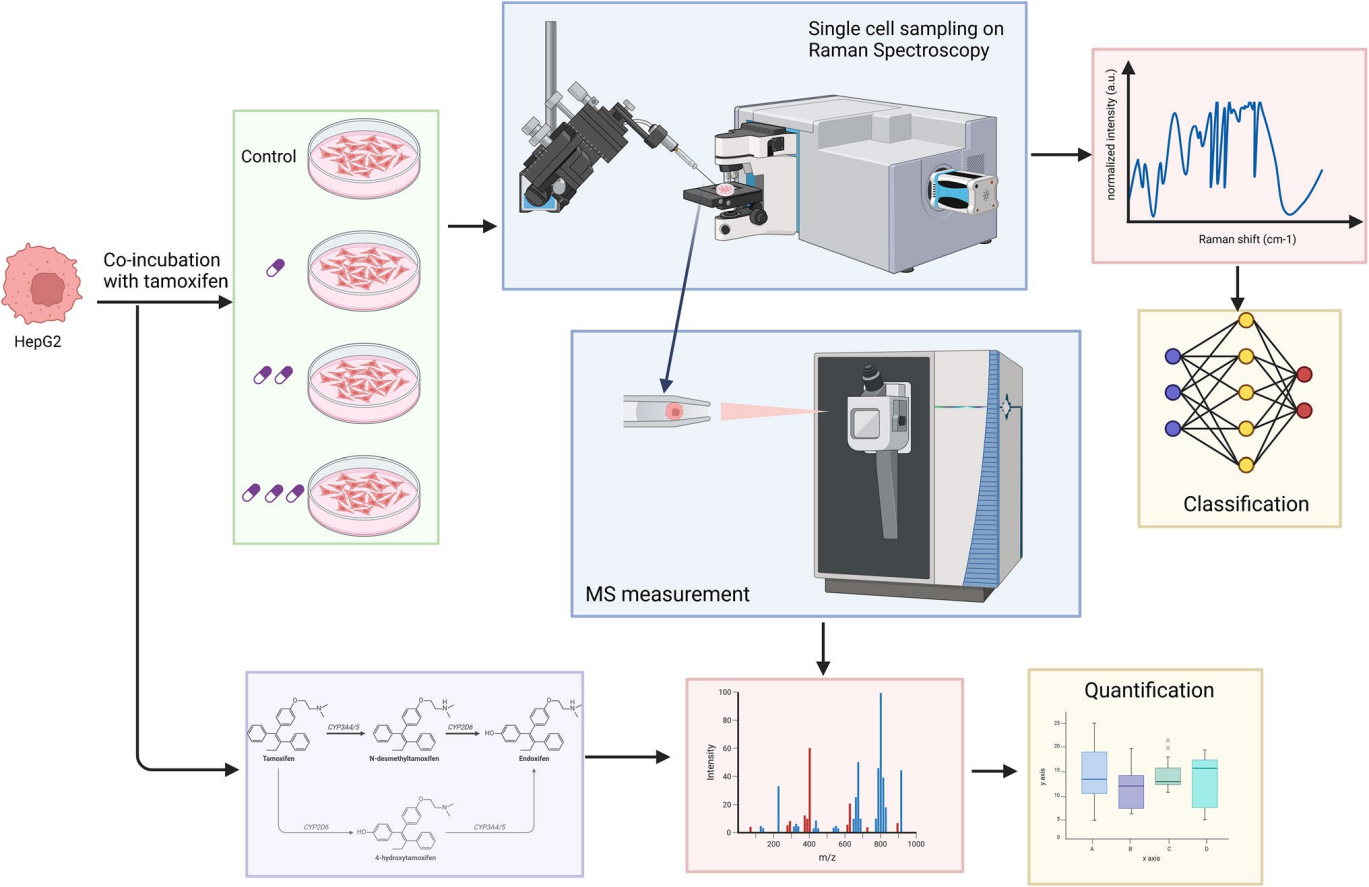
Schematic of the experimental setup used in this study

**Fig. 2:**
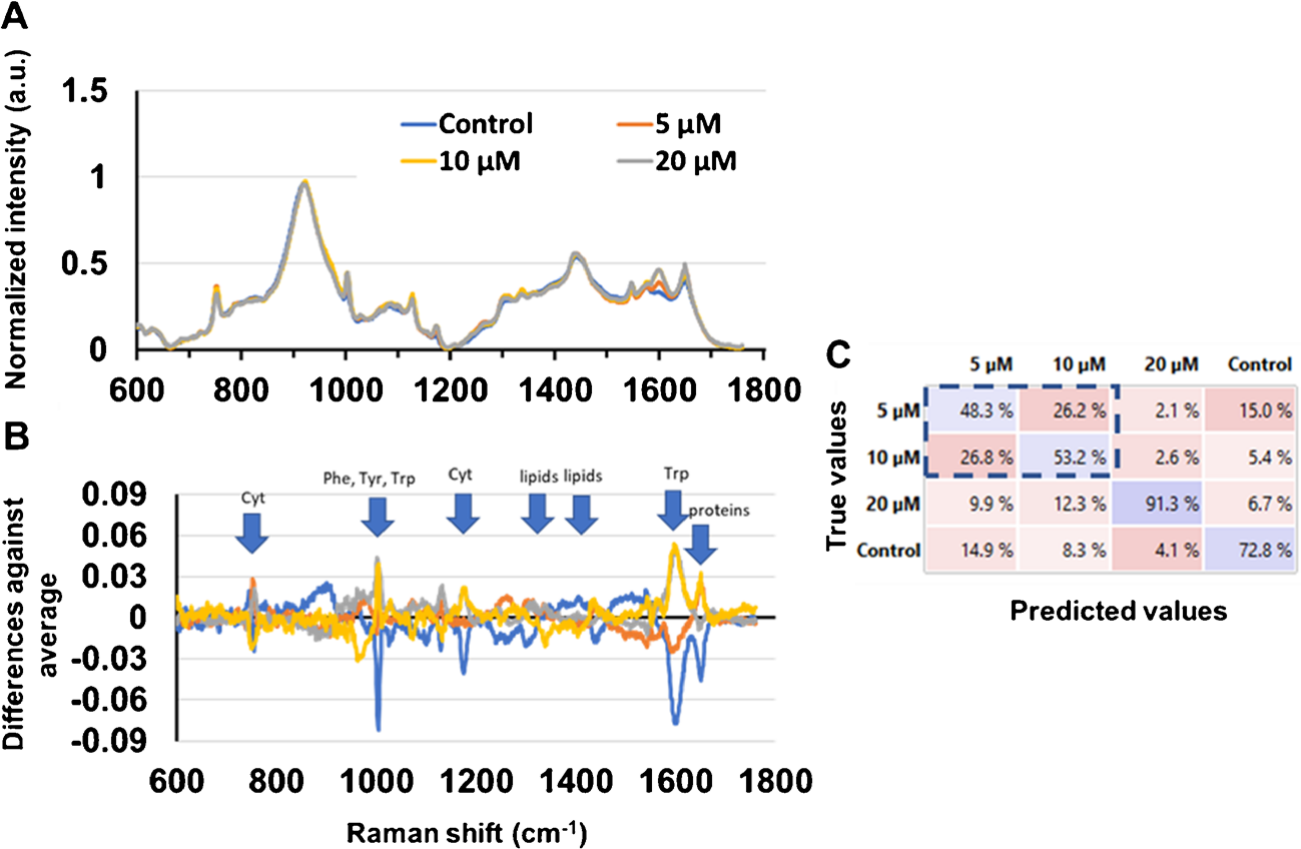
Spectral analysis of the fingerprint profile of single cells measured by Raman spectroscopy (n = 202). **A** Normalized Raman spectra. **B** Relative spectral differences of each group from the mean spectra of all the samples. **C** Confusion matrix obtained from the averaged prediction of the SVM models trained on spectral data. Results indicate that the prediction accuracy was strongly class-dependent

First, we explored the possibility of using non-destructive, label-free, confocal Raman spectroscopy to screen the effects of different concentrations of tamoxifen on single HepG2 cells (Fig. 1). The spectra of live single cells in the fingerprint region (~ 600 to ~ 1800 cm^−1^) are recorded. The averaged spectra of cells cultured at different concentrations of tamoxifen are plotted and are shown in Fig. 2A. The peak at 900 cm^−1^ represents the signal from the glass of the glass bottom dish, which served here as an internal reference point to normalize the spectra (0 to 1).

A visual comparison of normalized spectral intensities showed differences in the average spectral intensities under different concentrations of tamoxifen in the culture (Fig. 2A). In particular, at 20 µM, the signal shows several peaks with higher intensities than in other conditions. The relative spectral differences of each group compared to the average spectra were also compared in order to highlight the strongest differences across groups. Notably, the control group had a large difference to other groups for the peak at ~ 1000–1002 cm^−1^, associated with cyclic aromatic molecules, and the peak at 1603 and 1650 cm^−1^ associated with aromatic molecules and proteins (Fig. 2B).

Peaks with the largest difference in different groups were annotated based on previous studies (Table 1). The peak at ~ 752 cm^−1^, associated with cytochrome, and the peaks at ~ 1000–1002, 1173, 1403–1422, 1441–1450, 1603, 1650 cm^−1^ showed significant differences between different groups (ANOVA, Tukey HSD, p < 0.05). These peaks were identified as the important biomarkers for the drug activity based on their high Gini scores (Table 1).

As a means to demonstrate if spectral variations enable discrimination between the different treatments, a non-linear SVM model was computed. Compared with the true data, the predicted cells’ spectra were attributed to their correct type by 48.3% for the 5 µM drug concentration treatment. The remaining 5 µM treated cells were misidentified as control (14.8%) or 10 µM cells (26.2%) showing a close similarity of spectral signatures. On the other hand, the 20 µM treatment gave strong Raman shifts, which enable the SVM model to identify this group with a 91.3% accuracy. The model is thus confirming that metabolic shifts are strongly visible at a drug concentration of 20 µM. Control cells were identified with a 71% accuracy.

### Live single-cell MS (LSC-MS) measurement of tamoxifen, 4-OH-tamoxifen, and N-desmethyltamoxifen in single cells

Following Raman spectral analysis, single cells were picked up and analyzed by LSC-MS to measure tamoxifen and its metabolites’ abundance, namely 4-OH-tamoxifen and N-desmethyltamoxifen (Fig. 1).

**Fig. 3:**
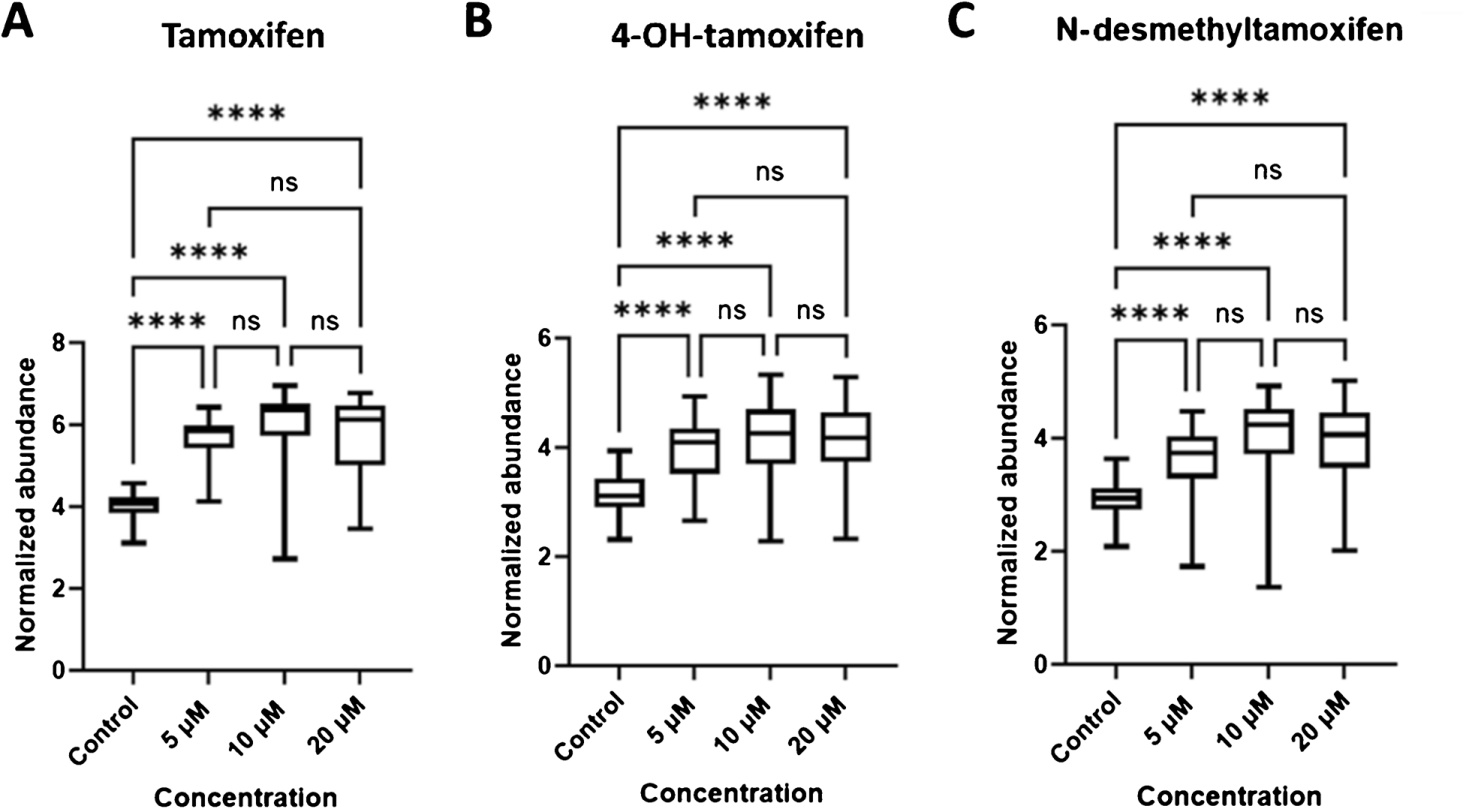
Distribution of tamoxifen, 4-OH-tamoxifen, and N-desmethyltamoxifen in single cells. Boxplot of **A** tamoxifen, **B** 4-OH-tamoxifen, and **C** N-desmethyltamoxifen normalized abundance values in different groups

Tamoxifen, 4-OH-tamoxifen, and N-desmethyltamoxifen could be detected in their [M + H]^+^ form (Fig. S-1). Figure 3 shows the relative abundance of tamoxifen and its metabolites among groups in single cells. The abundance increased with increasing concentration of the administered drug in the control, 5 μM and 10 μM groups. For tamoxifen, 4-OH-tamoxifen, and N-desmethyltamoxifen, the median abundance at the 20 μM group appears to level off or slightly decrease compared to the 10 μM group.

All the drug-treated groups show significant differences (ANOVA, Tukey HSD, p < 0.05) from the control group; however, we found non-significant differences between 5 μM and 10 μM and between 10 μM and 20 μM concentrations in the three drug-treated groups.

### Correlating Raman data and LSC-MS data

We subsequently explored whether the intensities of these peaks change in response to the concentration of tamoxifen.

**Fig. 4:**
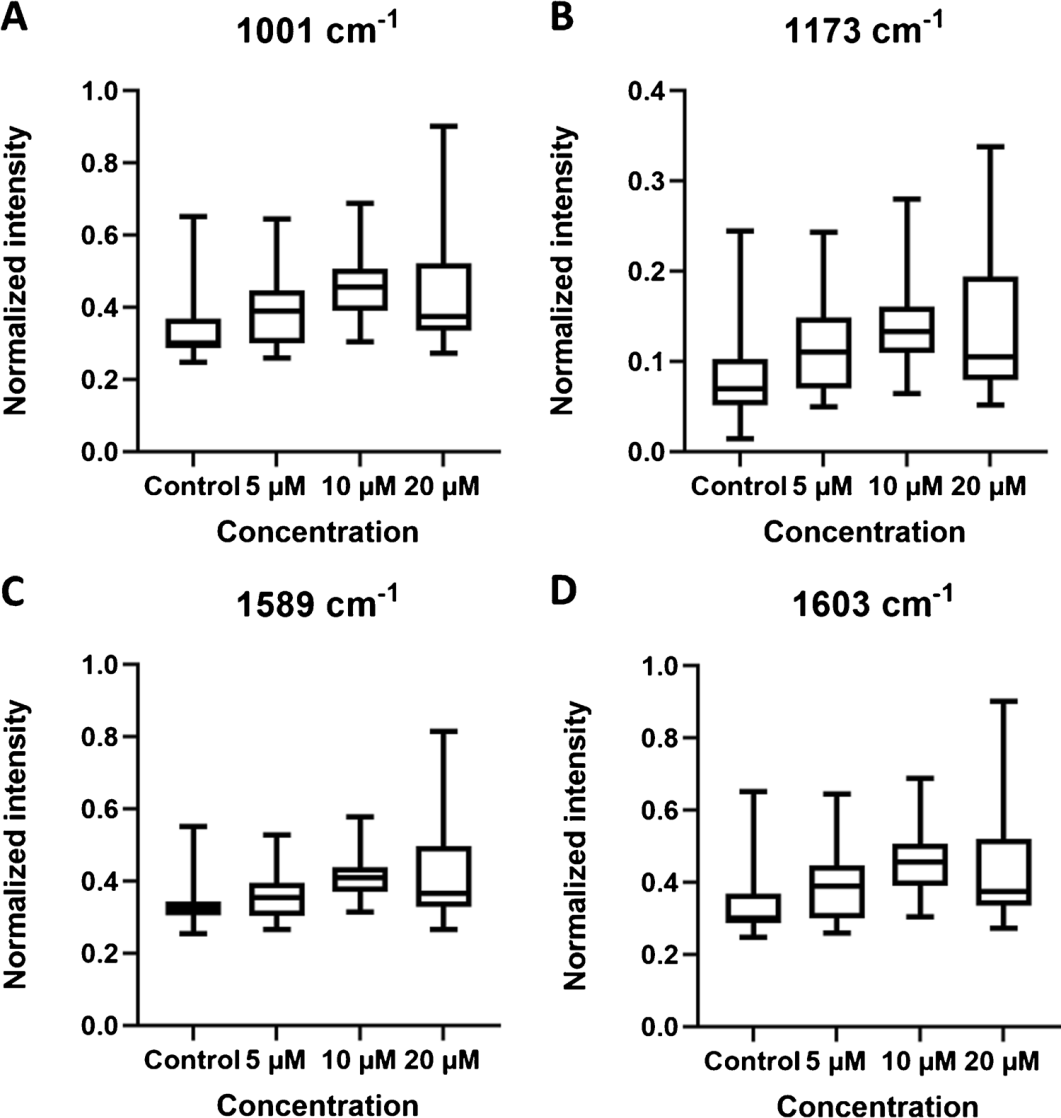
Intensity of Raman peaks at **A** 1001 cm^−1^, **B** 1173 cm^−1^, **C** 1589 cm^−1^, and **D** 1603 cm^−1^ in 4 treatment groups

Among the spectral peaks of interest highlighted in Fig. 2 and Table 1, a few were selected to be represented in Fig. 4. As can be seen in Fig. 4, the intensities of peaks 1001 cm^−1^, 1173 cm^−1^, 1589 cm^−1^, and 1603 cm^−1^ increased with the concentration of tamoxifen, but we found a decrease in the median for the 20 μM group compared to the 10 μM group, which is consistent with the trend of the MS data. Furthermore, we calculated the individual correlation between these four Raman shifts and tamoxifen and its two metabolites separately. The results showed a weak correlation (Figs. S−2, S-3, and S-4).

Finally, we also performed a correlation analysis (Fig. S-5) of the tamoxifen concentrations measured throughout the Raman spectra with the mass spectrometry. The direct correlation between Raman spectra and administered tamoxifen was weak.

## Discussion

It is currently unclear how the response of single cells is affected under varying degrees of drug exposure. To address this, our experiment aimed to examine the single-cell response of liver cancer cell line to incremental increases of tamoxifen concentrations in the media.

In our experiments, Raman fingerprinting showed that cells respond differently to different drug concentrations (Fig. 2). These differences suggest that tamoxifen at different concentrations results in altered spectral fingerprinting and that Raman could be used as a means of observing concentration-dependent drug-induced cellular metabolism. Furthermore, Raman spectroscopy could distinguish different groups based on Raman spectra (Fig. 2A), as well as identify key spectral biomarkers of the drug effect. On the other hand, LSC-MS revealed that both tamoxifen and its metabolites could be identified in single cells, as well as their relative abundance across the four measured groups; therefore, it revealed for the first time the concentration-dependent effect of tamoxifen on single cells in terms of drug uptake and metabolism.

Raman spectral analysis of single HepG2 cells treated with tamoxifen revealed differences across the multiple dosing concentrations, especially around certain wavelengths (Fig. 2A, Table 1). Multiple peaks were found to be elevated in the cells treated with 20 μM tamoxifen (752, 900, 1173, 1603, 1650 cm^−1^, Fig. 2). This can suggest these peaks are biomarkers of highly stressed cells or in the process of apoptosis due to the high tamoxifen dose. Okada and colleagues [35] showed that peaks 752 and 1133 cm^−1^ are associated with stronger cytochrome c activity. Cytochrome c plays a key role in mitochondrial endomembrane initiation, but early in the apoptosis process, the release of reactive oxygen species (ROS) from mitochondria triggers an oxidative reaction that leads to its release into the cytoplasm [36]. Since tamoxifen induces hepatotoxicity through apoptosis, we can hypothesize that the cell response at the highest tamoxifen concentration (20 μM) is linked to a stronger oxidative stress which stimulates cytochrome activity [37]. Moreover, the peaks at 1241, 1441 − 1450, and 1560 cm^−1^, which are associated with lipids, were chosen by the SVM model as important peaks to identify the 20 µM tamoxifen-spiked cell group. It has been shown that tamoxifen can interfere with hepatic lipid metabolism by inhibiting the degradation of fatty acids and the excretion of ApoB-100 [38]. Both these changes and the aforementioned cytochrome c peaks (752, 1133 cm^−1^) suggest that tamoxifen began to exert a noticeable effect on the cells starting from the 20 µM concentrations. Furthermore, it is important to note that despite the high level of confidence in biomarker identification afforded by Raman spectroscopy, additional orthogonal approaches might be needed to confirm the molecular structure of identified biomarkers.

The results of mass spectrometry experiments showed that we could detect and identify tamoxifen, 4-OH-tamoxifen, and N-desmethyltamoxifen (Fig. S-1). However, endoxifen, which is also a metabolite of tamoxifen, could not be detected. Since endoxifen requires a two-step reaction by cytochrome enzyme P450 to be produced [39], we hypothesize that endoxifen has a lower concentration in a single cell compared with the other two metabolites. This can explain why it could not be detected [40]. On the other hand, tamoxifen is metabolized to 4-OH-tamoxifen and N-desmethyltamoxifen in a one-step metabolic reaction, and tamoxifen, 4-OH-tamoxifen, and N-desmethyltamoxifen could be detected in single cells, as shown in Fig. 4. The overall trend in terms of the abundance of tamoxifen, 4-OH-tamoxifen, and N-desmethyltamoxifen across the different groups increased as the concentration of the administered drug increased, but there was a slight decrease when the concentration reached 20 μM. A possible reason for that is this concentration leads to cell apoptosis, and consequently, the cell activity or permeability was altered [41]. It has been shown that at a dosing concentration of up to 20 μM, all cells will have 40% cell survival after 24 h of co-incubation and 0% cell survival after 48 h [42], which demonstrates that at this concentration, cell permeability is altered due to apoptosis. The elevated cytochrome c and lipid-related peaks in 20 μM shown by the Raman data also support the hypothesis that a large number of cells undergo apoptosis at this concentration. This lends credence to the idea that there might be a threshold concentration between 10 and 20 μM of the drug from which cells cannot maintain their integrity after. This hypothesis of a threshold concentration is an interesting avenue of research that can be investigated in future studies.

In this study, the biological impact of different doses of tamoxifen as well as its metabolites’ relative abundance within the cells was revealed for the first time. However, this study is not without its limitations. At 20 µM, many cells were likely undergoing apoptosis, which impacted efforts to quantify the drug and its metabolite in single cells due to their possible leakage. Furthermore, it is important to note that Raman will likely have an effect on the cell metabolome. We attempted to mitigate this by sampling and quenching the cell metabolism immediately after irradiation. However, the exact effects of the Raman laser must be accounted for in untargeted metabolic experiments. This can be achieved by measuring cells that were irradiated and comparing them with unirradiated cells as controls. Meanwhile, the lower throughput of mass spectrometry measurements is a limitation that needs to be overcome, especially when combined with the high-throughput Raman spectroscopy systems [32]. An additional advantage of increased throughput is the ability to discern possible subpopulations in drug-treated cells that could not be identified before. Therefore, the development of a higher throughput single-cell mass spectrometry method is essential. Finally, correlating the two datasets obtained from the different techniques remains a challenge. Using an internal standard that can be detected in both methods can be a way to address this. Despite these limitations, our method showed the extent and the impact of heterogeneity on cellular drug uptake and metabolism, as well as the drug effect. We hope that this study will encourage further questioning of the mechanism that could be responsible for the threshold effect of the drug concentration in cells as well as the linearity of drug response on the single-cell level. Moreover, functional heterogeneity at the cellular level may exert a substantial influence on therapeutic efficacy and the emergence of drug resistance within clinical contexts, particularly in treatment protocols that incorporate targeted agents or are dependent on defined metabolic pathways. To answer such questions, the development of an integrated platform capable of monitoring single-cell pharmacokinetics and pharmacodynamics will be required. Having established the proposed technology as a viable platform for drug monitoring studies, we aim to further advance its capabilities to enable direct probing of intracellular contents without the need for cellular extraction. We believe that the combination of techniques such as label-free chemical imaging, MS, and machine learning is one promising way of achieving this goal.

## Supplementary information

Below is the link to the electronic supplementary material.

ESM1 (PPTX 1.01 MB)

## Data Availability

All data generated or analyzed during this study are included in this published article. Further details are available from the corresponding author on reasonable request.

## References

[1] Bunnage ME, Chekler ELP, Jones LH. Target validation using chemical probes. Nat Chem Biol. 2013;9:195–9. 10.1038/nchembio.1197. 23508172 10.1038/nchembio.1197

[2] Zhang A, Zou J, Xi Y, Gao L, Deng F, Liu Y, et al. Single-cell technology for drug discovery and development. Front Drug Discov. 2024;4:1459962. 10.3389/fddsv.2024.1459962.

[3] Zenobi R. Single-cell metabolomics: analytical and biological perspectives. Science. 2013;342:1243259. 10.1126/science.1243259. 24311695 10.1126/science.1243259

[4] Altschuler SJ, Wu LF. Cellular heterogeneity: do differences make a difference? Cell. 2010;141:559–63. 10.1016/j.cell.2010.04.033. 20478246 10.1016/j.cell.2010.04.033 PMC2918286

[5] Amir ED, Davis KL, Tadmor MD, Simonds EF, Levine JH, Bendall SC, et al. viSNE enables visualization of high dimensional single-cell data and reveals phenotypic heterogeneity of leukemia. Nat Biotechnol. 2013;31:545–52. 10.1038/nbt.2594. 23685480 10.1038/nbt.2594 PMC4076922

[6] Zhang C, Le Dévédec SE, Ali A, Hankemeier T. Single-cell metabolomics by mass spectrometry: ready for primetime? Curr Opin Biotechnol. 2023;82: 102963. 10.1016/j.copbio.2023.102963. 37356380 10.1016/j.copbio.2023.102963

[7] Wang Y, Wang Y, Lü J, Li X. Unraveling the drug response heterogeneity with single-cell vibrational phenomics. Cell Biochem Biophys. 2024;82:2503–10. 10.1007/s12013-024-01363-0. 38914839 10.1007/s12013-024-01363-0

[8] Yang Y, Mansfeld FM, Kavallaris M, Gaus K, Tilley RD, Gooding JJ. Monitoring the heterogeneity in single cell responses to drugs using electrochemical impedance and electrochemical noise. Chem Sci. 2021;12:2558–66. 10.1039/d0sc05489e. 10.1039/d0sc05489e PMC8179273 34164023

[9] Lee M-CW, Lopez-Diaz FJ, Khan SY, Tariq MA, Dayn Y, Vaske CJ, et al. Single-cell analyses of transcriptional heterogeneity during drug tolerance transition in cancer cells by RNA sequencing. Proc Natl Acad Sci USA. 2014. 10.1073/pnas.1404656111. 25339441 10.1073/pnas.1404656111 PMC4226127

[10] Ianevski A, Nader K, Driva K, Senkowski W, Bulanova D, Moyano-Galceran L, et al. Single-cell transcriptomes identify patient-tailored therapies for selective co-inhibition of cancer clones. Nat Commun. 2024;15:8579. 10.1038/s41467-024-52980-5. 39362905 10.1038/s41467-024-52980-5 PMC11450203

[11] Kim K-T, Lee HW, Lee H-O, Kim SC, Seo YJ, Chung W, et al. Single-cell mRNA sequencing identifies subclonal heterogeneity in anti-cancer drug responses of lung adenocarcinoma cells. Genome Biol. 2015;16:127. 10.1186/s13059-015-0692-3. 26084335 10.1186/s13059-015-0692-3 PMC4506401

[12] Kim K-T, Lee HW, Lee H-O, Song HJ, Jeong DE, Shin S, et al. Application of single-cell RNA sequencing in optimizing a combinatorial therapeutic strategy in metastatic renal cell carcinoma. Genome Biol. 2016;17:80. 10.1186/s13059-016-0945-9. 27139883 10.1186/s13059-016-0945-9 PMC4852434

[13] Li M, Liao H-X, Bando K, Nawa Y, Fujita S, Fujita K. Label-free monitoring of drug-induced cytotoxicity and its molecular fingerprint by live-cell Raman and autofluorescence imaging. Anal Chem. 2022;94:10019–26. 10.1021/acs.analchem.2c00293. 35786862 10.1021/acs.analchem.2c00293

[14] Huser T, Chan J. Raman spectroscopy for physiological investigations of tissues and cells. Adv Drug Deliv Rev. 2015;89:57–70. 10.1016/j.addr.2015.06.011. 26144996 10.1016/j.addr.2015.06.011

[15] Lee YJ, Vega SL, Patel PJ, Aamer KA, Moghe PV, Cicerone MT. Quantitative, label-free characterization of stem cell differentiation at the single-cell level by broadband coherent anti-Stokes Raman scattering microscopy. Tissue Eng Part C Methods. 2014;20:562–9. 10.1089/ten.tec.2013.0472. 24224876 10.1089/ten.tec.2013.0472 PMC4074749

[16] Germond A, Panina Y, Shiga M, Niioka H, Watanabe TM. Following embryonic stem cells, their differentiated progeny, and cell-state changes during iPS reprogramming by Raman spectroscopy. Anal Chem. 2020;92:14915–23. 10.1021/acs.analchem.0c01800. 33112148 10.1021/acs.analchem.0c01800

[17] Beton-Mysur K, Kopec M, Brozek-Pluska B. Raman imaging—a valuable tool for tracking fatty acid metabolism—normal and cancer human colon single-cell study. IJMS. 2024;25:4508. 10.3390/ijms25084508. 38674093 10.3390/ijms25084508 PMC11050638

[18] Buckmaster R, Asphahani F, Thein M, Xu J, Zhang M. Detection of drug-induced cellular changes using confocal raman spectroscopy on patterned single-cell biosensors. Analyst. 2009;134:1440. 10.1039/b900420c. 19562213 10.1039/b900420c PMC2902718

[19] Owen CA, Selvakumaran J, Notingher I, Jell G, Hench LL, Stevens MM. In vitro toxicology evaluation of pharmaceuticals using raman micro-spectroscopy. J of Cellular Biochemistry. 2006;99:178–86. 10.1002/jcb.20884. 10.1002/jcb.20884 16598770

[20] Parachalil DR, Commerford D, Bonnier F, Chourpa I, McIntyre J, Byrne HJ. Raman spectroscopy as a potential tool for label free therapeutic drug monitoring in human serum: the case of busulfan and methotrexate. Analyst. 2019;144:5207–14. 10.1039/c9an00801b. 31355390 10.1039/c9an00801b

[21] Ali A, Abouleila Y, Shimizu Y, Hiyama E, Watanabe TM, Yanagida T, et al. Single-cell screening of tamoxifen abundance and effect using mass spectrometry and raman-spectroscopy. Anal Chem. 2019;91:2710–8. 10.1021/acs.analchem.8b04393. 30664349 10.1021/acs.analchem.8b04393

[22] Nakatani K, Izumi Y, Hata K, Bamba T. An analytical system for single-cell metabolomics of typical mammalian cells based on highly sensitive nano-liquid chromatography tandem mass spectrometry. Mass Spectrom. 2020;9: A0080–A0080. 10.5702/massspectrometry.A0080. 10.5702/massspectrometry.A0080 PMC7242784 32547894

[23] Ali A, Abouleila Y, Shimizu Y, Hiyama E, Emara S, Mashaghi A, et al. Single-cell metabolomics by mass spectrometry: advances, challenges, and future applications. TrAC Trends Anal Chem. 2019;120:115436. 10.1016/j.trac.2019.02.033.

[24] Martens J, Koppen V, Berden G, Cuyckens F, Oomens J. Combined liquid chromatography-infrared ion spectroscopy for identification of regioisomeric drug metabolites. Anal Chem. 2017;89:4359–62. 10.1021/acs.analchem.7b00577. 28368097 10.1021/acs.analchem.7b00577 PMC5397882

[25] Lanni EJ, Masyuko RN, Driscoll CM, Dunham SJB, Shrout JD, Bohn PW, et al. Correlated imaging with C _60_ -SIMS and confocal raman microscopy: visualization of cell-scale molecular distributions in bacterial biofilms. Anal Chem. 2014;86:10885–91. 10.1021/ac5030914. 25268906 10.1021/ac5030914 PMC4221875

[26] Germond A, Ichimura T, Horinouchi T, Fujita H, Furusawa C, Watanabe TM. Raman spectral signature reflects transcriptomic features of antibiotic resistance in *Escherichia coli*. Commun Biol. 2018;1:85. 10.1038/s42003-018-0093-8. 30271966 10.1038/s42003-018-0093-8 PMC6123714

[27] Gold C, Sollich P. Model selection for support vector machine classification. Neurocomputing. 2003;55:221–49. 10.1016/S0925-2312(03)00375-8.

[28] Zhang J, Lin H, Xu J, Zhang M, Ge X, Zhang C, et al. High-throughput single-cell sorting by stimulated Raman-activated cell ejection. 2023. 10.1101/2023.10.16.562526. 10.1126/sciadv.adn6373 PMC11633747 39661682

[29] Demšar J, Curk T, Erjavec A, Gorup Č, Hočevar T, Milutinovič M, et al. Orange: data mining toolbox in Python. The Journal of Machine Learning Research. 2013;14:2349–53.

[30] Pezzotti G. Raman spectroscopy in cell biology and microbiology. J Raman Spectrosc. 2021;52:2348–443. 10.1002/jrs.6204.

[31] Lasalvia M, Scrima R, Perna G, Piccoli C, Capitanio N, Biagi PF, et al. Exposure to 1.8 GHz electromagnetic fields affects morphology, DNA-related raman spectra and mitochondrial functions in human lympho-monocytes. PLoS One. 2018;13: e0192894. 10.1371/journal.pone.0192894. 29462174 10.1371/journal.pone.0192894 PMC5819811

[32] Allakhverdiev ES, Khabatova VV, Kossalbayev BD, Zadneprovskaya EV, Rodnenkov OV, Martynyuk TV, et al. Raman spectroscopy and its modifications applied to biological and medical research. Cells. 2022;11:386. 10.3390/cells11030386. 35159196 10.3390/cells11030386 PMC8834270

[33] Depciuch J, Jakubczyk P, Paja W, Pancerz K, Wosiak A, Kula-Maximenko M, et al. Correlation between human colon cancer specific antigens and Raman spectra. Attempting to use Raman spectroscopy in the determination of tumor markers for colon cancer. Nanomed Nanotechnol Biol Med. 2023;48: 102657. 10.1016/j.nano.2023.102657. 10.1016/j.nano.2023.102657 36646194

[34] Zhu N, Wu D, Chen K. Label-free visualization of fruit lignification: raman molecular imaging of loquat lignified cells. Plant Methods. 2018;14:58. 10.1186/s13007-018-0328-1. 30008794 10.1186/s13007-018-0328-1 PMC6043974

[35] Okada M, Smith NI, Palonpon AF, Endo H, Kawata S, Sodeoka M, et al. Label-free raman observation of cytochrome c dynamics during apoptosis. Proc Natl Acad Sci U S A. 2012;109:28–32. 10.1073/pnas.1107524108. 22184220 10.1073/pnas.1107524108 PMC3252932

[36] Kulikov AV, Shilov ES, Mufazalov IA, Gogvadze V, Nedospasov SA, Zhivotovsky B. Cytochrome c: the achilles’ heel in apoptosis. Cell Mol Life Sci. 2012;69:1787–97. 10.1007/s00018-011-0895-z. 22179840 10.1007/s00018-011-0895-z PMC11114681

[37] Ahmed NS, Samec M, Liskova A, Kubatka P, Saso L. Tamoxifen and oxidative stress: an overlooked connection. Discover Oncol. 2021;12: 17. 10.1007/s12672-021-00411-y. 10.1007/s12672-021-00411-y PMC8777555 35201439

[38] Krähenbühl S. Effect of toxicants on fatty acid metabolism in HepG2 cells. Front Pharmacol. 2018. 10.3389/fphar.2018.00257. 29740314 10.3389/fphar.2018.00257 PMC5924803

[39] EH-Haj BM. Metabolic n-dealkylation and n-oxidation as elucidators of the role of alkylamino moieties in drugs acting at various receptors. Molecules. 2021;26: 1917. 10.3390/molecules26071917. 33805491 10.3390/molecules26071917 PMC8036657

[40] Archid R, Reymond M, Wilson R. Reprogramming of mesothelial-mesenchymal transition in chronic peritoneal diseases by estrogen receptor modulation and TGF-β1 inhibition. Int J Mol Sci. 2020. 10.3390/ijms21114158. 32532126 10.3390/ijms21114158 PMC7312018

[41] Sanaei M, Kavoosi F, Atashpour S, Haghighat S. Effects of genistein and synergistic action in combination with tamoxifen on the HepG2 human hepatocellular carcinoma cell line. Asian Pac J Cancer Prev. 2017;18:2381–5. 10.22034/APJCP.2017.18.9.2381. 28950682 10.22034/APJCP.2017.18.9.2381 PMC5720640

[42] Brandt S, Heller H, Schuster K, Grote J. Tamoxifen induces suppression of cell viability and apoptosis in the human hepatoblastoma cell line HepG2 via down-regulation of telomerase activity. Liver Int. 2004;24:46–54. 10.1111/j.1478-3231.2004.00887.x. 15102000 10.1111/j.1478-3231.2004.00887.x

