# Supplementary material for: Single-cell Raman and mass spectrometry analysis to probe cellular heterogeneity in tamoxifen uptake and metabolism
Source: Anal Bioanal Chem. 2025 Aug 14;417(23):5349–58. doi: 10.1007/s00216-025-06058-w (PMC12431888; doi:10.1007/s00216-025-06058-w)
Supplement: Supplementary file 1 — (PPTX 1.01 MB) [file 216_2025_6058_MOESM1_ESM.pptx]

## Slide 1
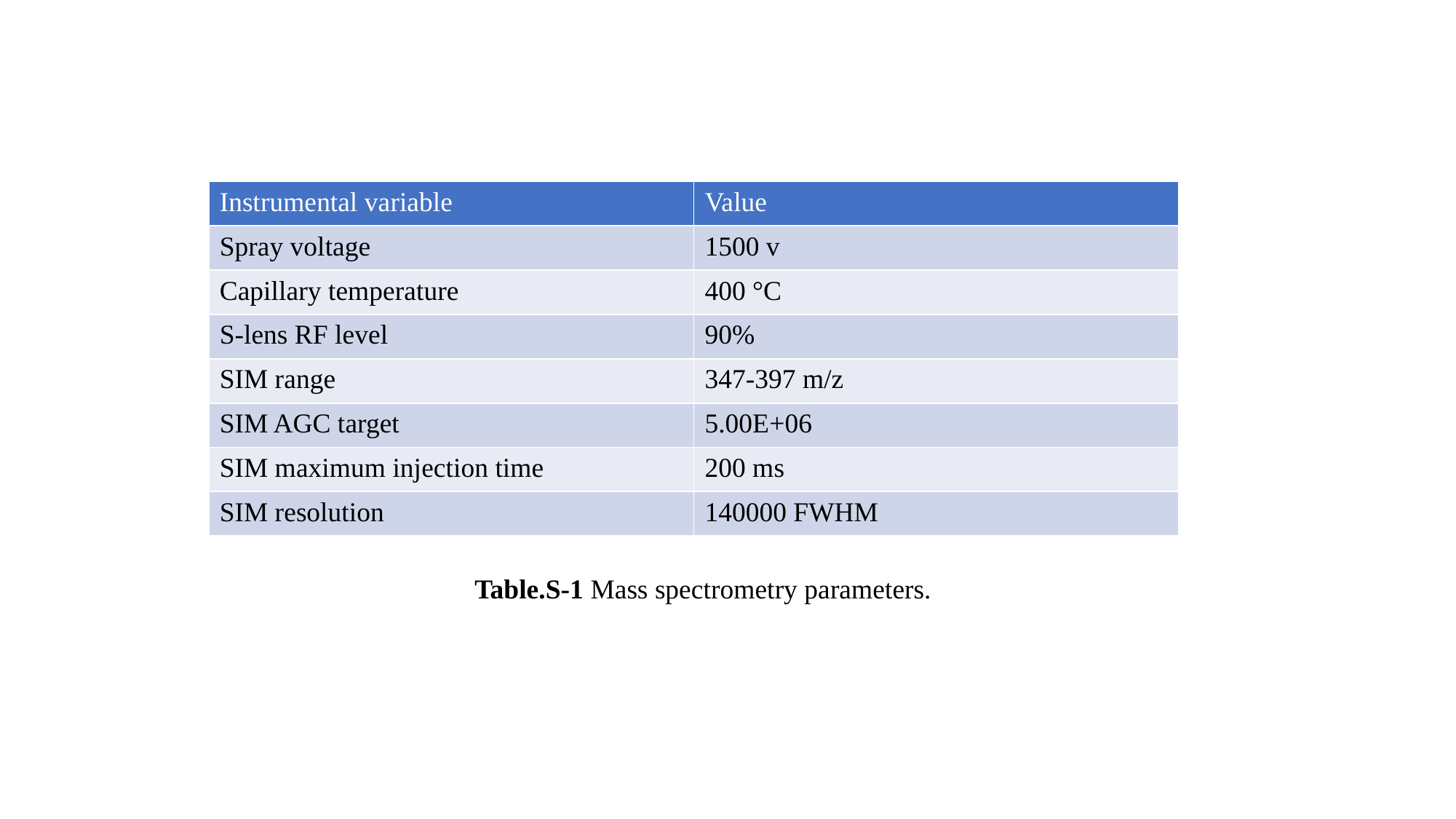

| Instrumental variable | Value |
| --- | --- |
| Spray voltage | 1500 v |
| Capillary temperature | 400 °C |
| S-lens RF level | 90% |
| SIM range | 347-397 m/z |
| SIM AGC target | 5.00E+06 |
| SIM maximum injection time | 200 ms |
| SIM resolution | 140000 FWHM |
Table.S-1 Mass spectrometry parameters.

## Slide 2
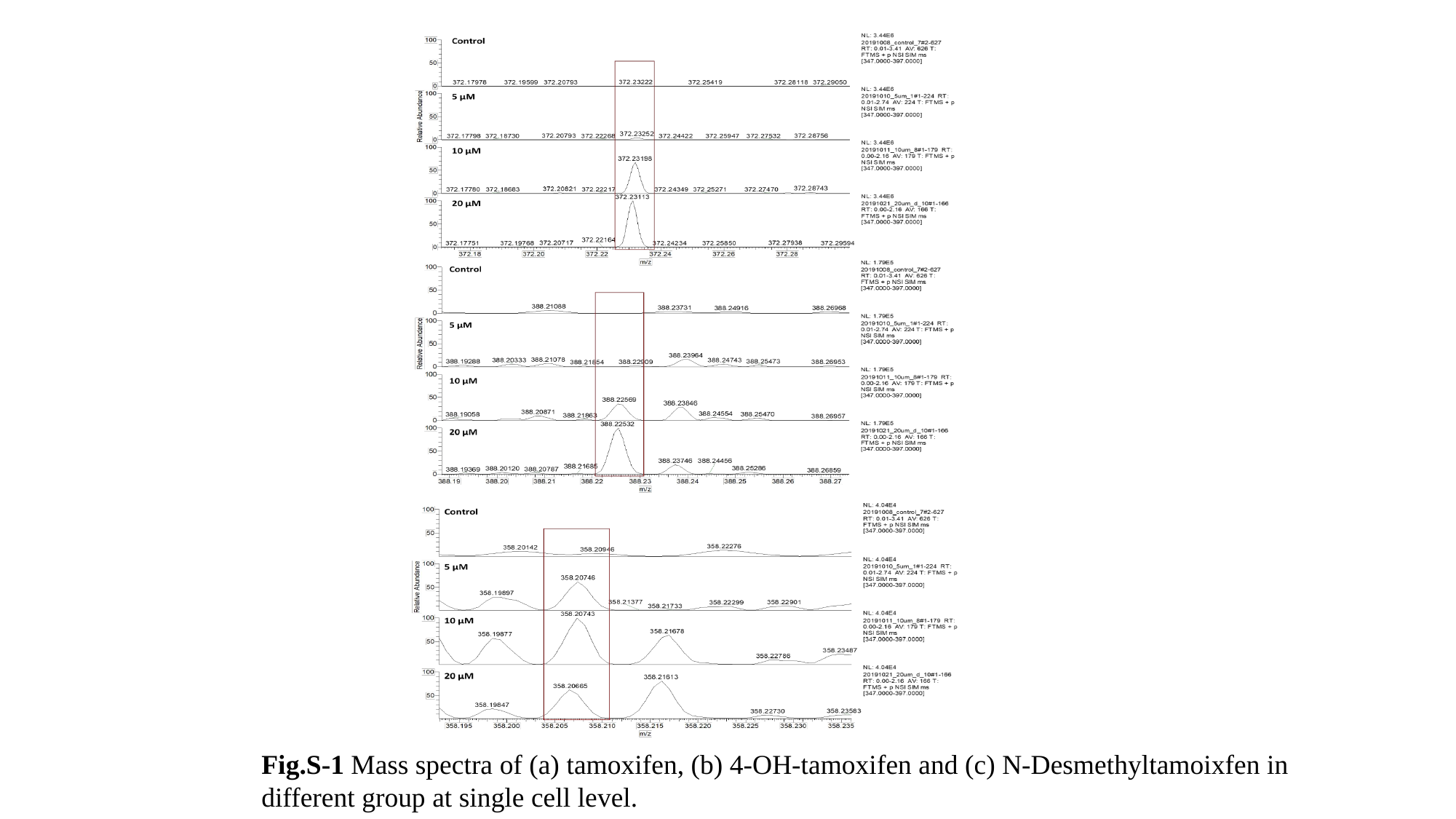

Fig.S-1 Mass spectra of (a) tamoxifen, (b) 4-OH-tamoxifen and (c) N-Desmethyltamoixfen in different group at single cell level.

## Slide 3
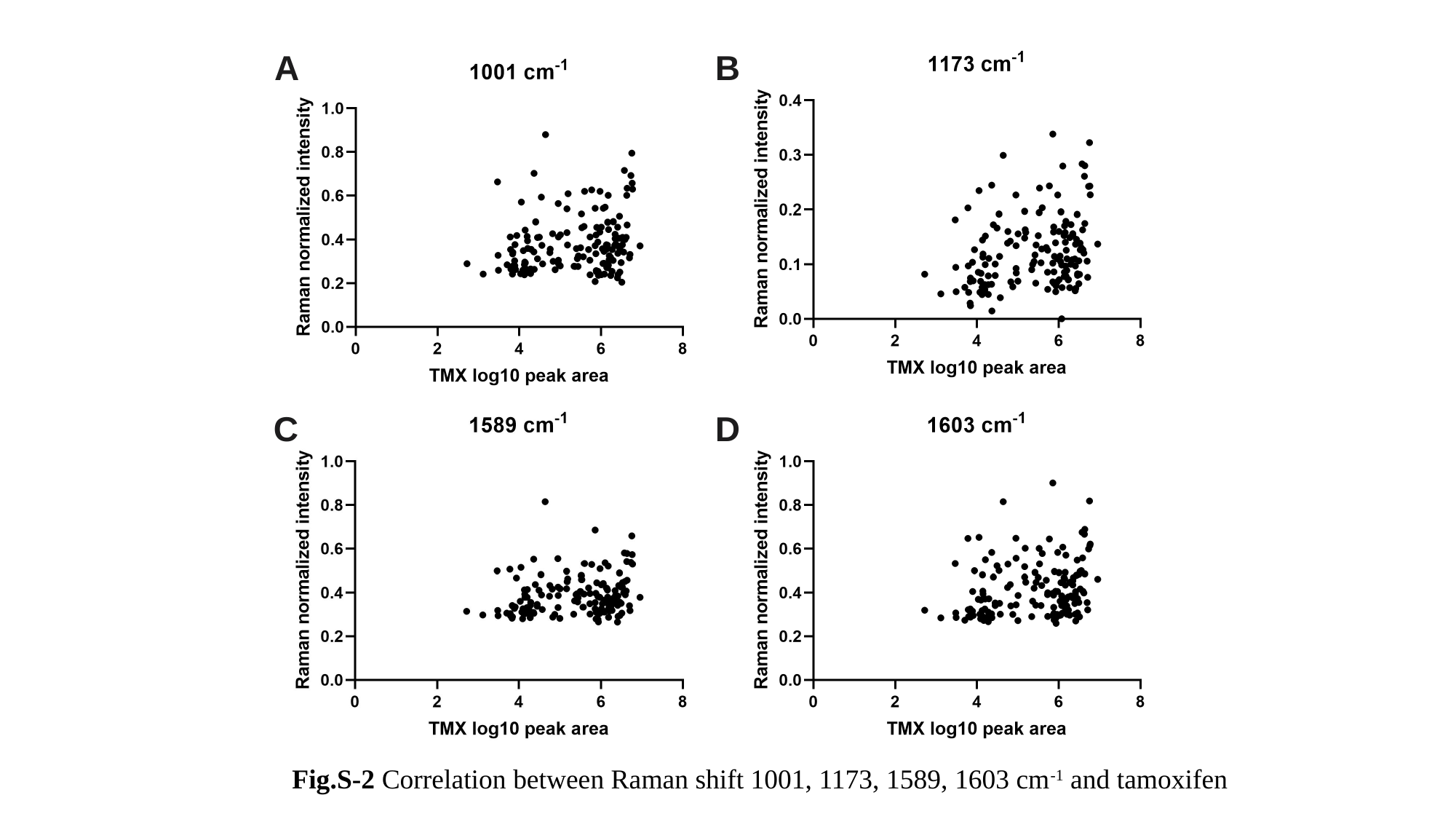

B
A
C
D
Fig.S-2 Correlation between Raman shift 1001, 1173, 1589, 1603 cm-1 and tamoxifen

## Slide 4
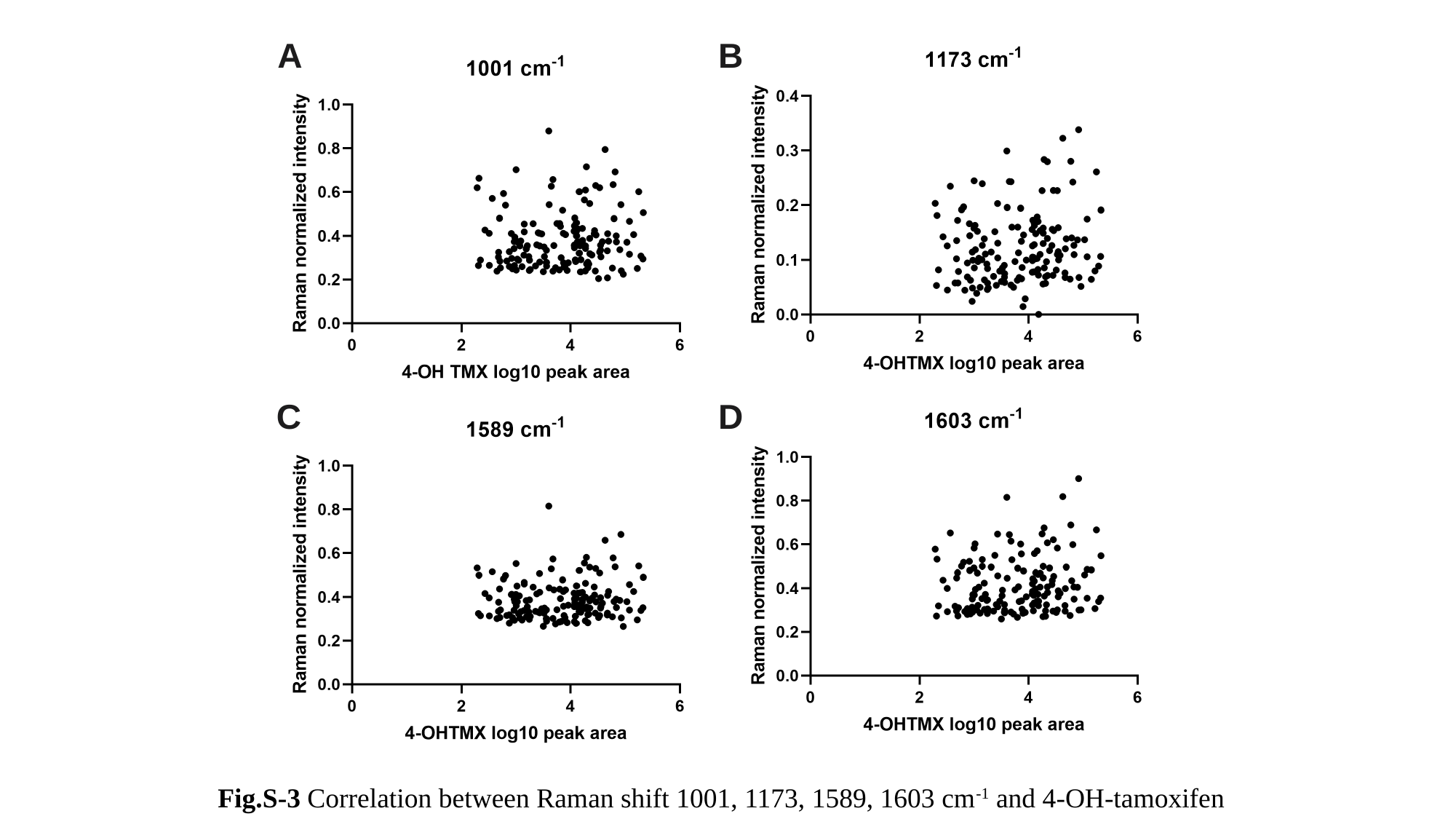

B
A
C
D
Fig.S-3 Correlation between Raman shift 1001, 1173, 1589, 1603 cm-1 and 4-OH-tamoxifen

## Slide 5
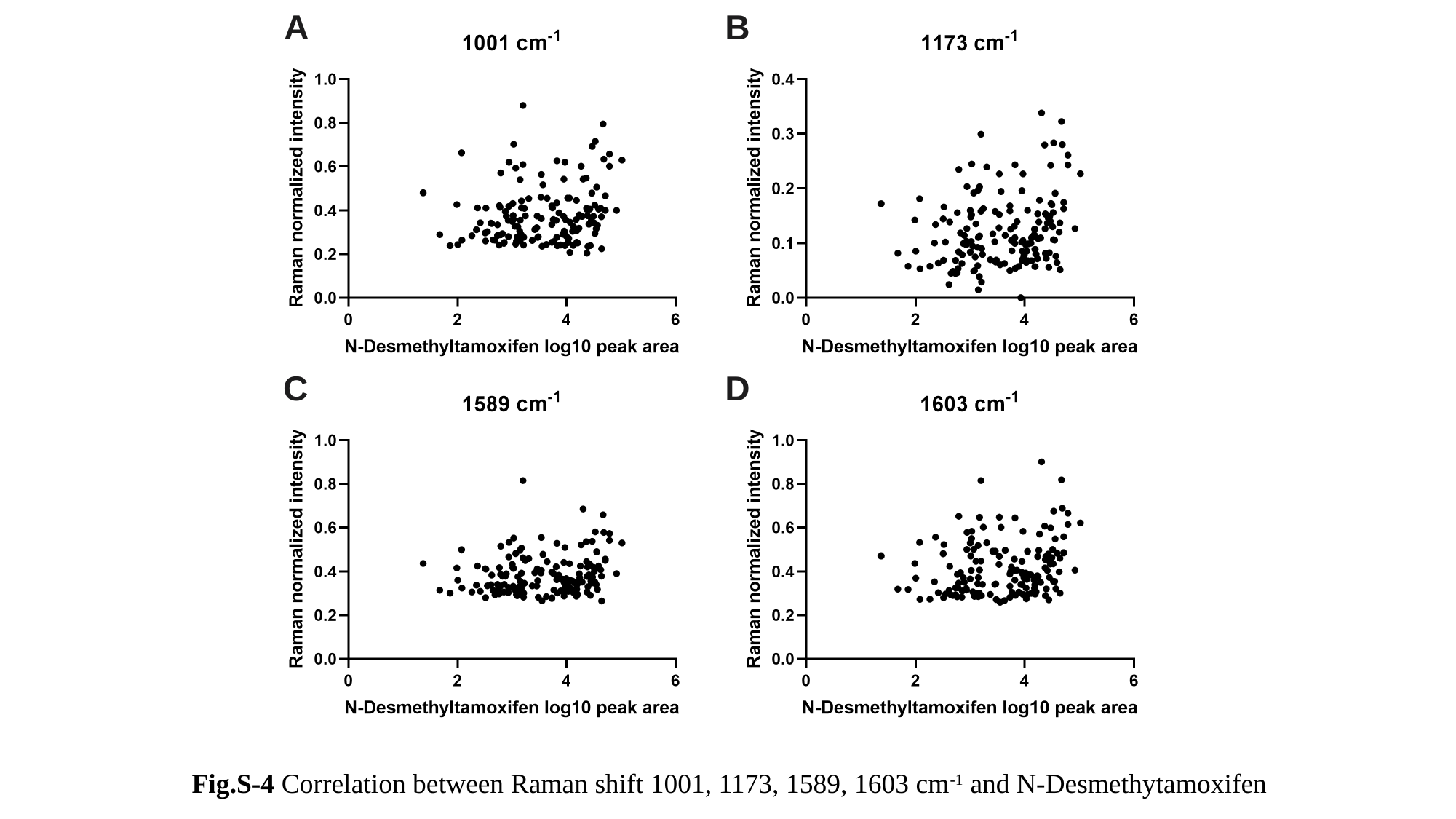

B
A
C
D
Fig.S-4 Correlation between Raman shift 1001, 1173, 1589, 1603 cm-1 and N-Desmethytamoxifen

## Slide 6
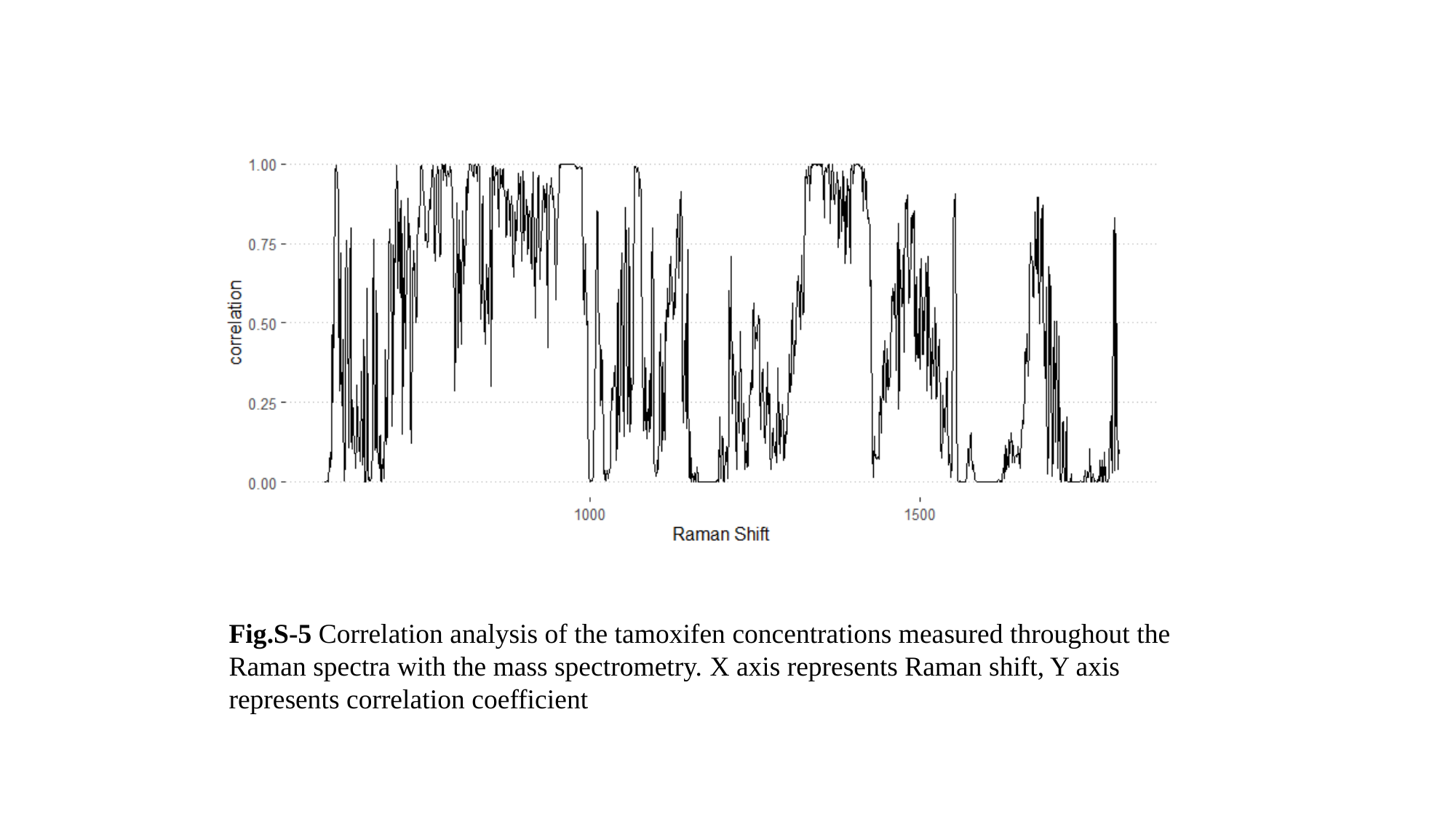

Fig.S-5 Correlation analysis of the tamoxifen concentrations measured throughout the Raman spectra with the mass spectrometry. X axis represents Raman shift, Y axis represents correlation coefficient
